# Emotional Valence Affects Word Retrieval During Verb Fluency Tasks in Alzheimer’s Dementia

**DOI:** 10.3389/fpsyg.2021.777116

**Published:** 2021-12-02

**Authors:** Eun Jin Paek

**Affiliations:** Department of Audiology and Speech Pathology, College of Health Professions, The University of Tennessee Health Science Center, Knoxville, TN, United States

**Keywords:** Alzheimer’s disease, dementia, emotional enhancement of memory, emotional valence, word retrieval, verb fluency, action fluency

## Abstract

Individuals with amnestic Alzheimer’s disease (AD) often demonstrate preserved emotional processing skills despite the neurodegenerative disease that affects their limbic system. Emotional valence encompasses the encoding and retrieval of memory and it also affects word retrieval in healthy populations, but it remains unclear whether these effects are preserved in individuals with amnestic AD. Previous studies used a variety of encoding procedures and different retrieval methods that resulted in mixed findings. Therefore, the purpose of the current study is to investigate whether emotional enhancement of memory effects is observed in an experimental condition where the memory encoding process is not required, namely verb (action) fluency tasks. Seventeen participants who were cognitively healthy older adults (CHOA) and 15 participants with amnestic AD were asked to complete verb fluency tasks, and the relative degree of emotional valence observed in their responses was compared between the two groups. A neuropsychological test battery was administered to determine the participants’ cognitive and linguistic profiles, and correlational analyses were conducted to delineate relationships between emotional valence, verbal memory, and learning abilities. The results indicated that the participants with amnestic AD produced words with higher emotional valence (i.e., more pleasant words) compared to CHOA during action fluency testing. In addition, the degree of emotional valence in the words was negatively correlated with verbal memory and learning skills, showing that those with poorer memory skills tend to retrieve words with higher emotional valence. The findings are consistent with those previous studies that stressed that individuals with AD have preserved emotional enhancement of memory effects and may benefit from them for retrieval of information, which may offer some insight into the development of novel rehabilitative strategies for this population.

## Introduction

Alzheimer’s disease (AD) and Alzheimer’s disease-related dementias (ADRDs) represent a worldwide public health crisis that affects the daily lives of those with dementia, presents significant and substantial challenges to their caregivers, and results in a considerable cost of care (Alzheimer’s Association, 2021). Those with AD may exhibit different phenotypes of dementia, although the most commonly observed phenotype involves substantial impairment of memory (McKhann et al., 2011), namely amnestic AD. Individuals with amnestic AD often exhibit breakdowns in communication skills and progressive declines in memory and other cognitive changes, which can lead to decreased social activity and engagement and to increased distress in both patients and caregivers (Kavé and Goral, 2016; Alzheimer’s Association, 2021). Furthermore, the constellation of cognitive and linguistic symptoms may prevent patients with AD from implementing strategies to control or decrease the risk of further cognitive declines, such as active engagement in cognitive, social, and leisure activities (Di Marco et al., 2014; Yates et al., 2016).

Although no definitive cure for AD has been developed, some progress has been made in understanding the cognitive and communication disorders exhibited by individuals with AD and to determine facilitative strategies to maintain or maximize their remaining cognitive and communication skills. For example, whereas episodic and semantic memory breakdowns are observed in individuals with AD from early in the course of the disease, implicit memory is relatively well preserved in earlier stages of the disease (Harrison et al., 2007; De Wit et al., 2021). Accordingly, management and treatment approaches capitalize on these relatively intact cognitive domains that are less affected by the neuropathological changes in AD, such as spaced retrieval training (Oren et al., 2014).

In addition to implicit memory being relatively preserved until later in the disease, the effect of emotion on memory in AD might hold potential in development of intervention and management strategies, particularly because some emotional processing skills also remain relatively intact in AD (Labar et al., 2005; Blessing et al., 2006; Broster et al., 2012; Perrin et al., 2012; Baran et al., 2014; Guzmán-Vélez et al., 2014; Giffard et al., 2015; Gomez-Gallego and Gomez-Garcia, 2017; van Dulmen et al., 2017). For example, individuals with AD often exhibit feelings without memory, anecdotally reporting that they remember how they felt during a conversation, although they do not recall what was discussed, when it happened, or even with whom they spoke. Consistent with anecdotal reports, research literature indicates that emotional processes are actually better preserved and stored than explicit or declarative memory in AD (Guzmán-Vélez et al., 2014). In addition, individuals with AD showed no differences compared to healthy older adults when rating or recognizing emotion in images or words (Labar et al., 2005; Perrin et al., 2012; Baran et al., 2014; Gomez-Gallego and Gomez-Garcia, 2017). Furthermore, emotion in AD seems to affect significantly the patients’ memory formation and behaviors, at least during the earlier stages of disease progression (Wright et al., 2007; Nashiro and Mather, 2011; Klein-Koerkamp et al., 2012; Perrin et al., 2012; Kalenzaga et al., 2015).

Emotional valence refers to how pleasant a stimulus is and it can be measured at the lexical level to characterize degrees of emotion in a word (Warriner et al., 2013; Kuperman et al., 2014; Kauschke et al., 2019). Emotional valence ratings range from 1 to 9, with a higher value indicating more pleasantness or happiness. For example, the emotional valence of “dance” is 7.27, while that of “kneel” is 4. When information is presented to or retrieved from individuals with AD in the context or with the content of positive or negative emotion, there exist facilitatory effects of emotion on memory formation (retrieval or recognition) and word processing (Labar et al., 2005; Brueckner and Moritz, 2009 Sundstrøm, 2011; Perrin et al., 2012; Giffard et al., 2015; Sava et al., 2015; Bohn et al., 2016; Gomez-Gallego and Gomez-Garcia, 2017; Rodríguez-Ferreiro et al., 2019). For example, individuals with mild AD were better able to recall the gist of memory when the information was provided with positive or negative emotions versus neutral context (Perrin et al., 2012).

To date, however, findings on the effects of emotional valence on memory processes in AD have been somewhat divergent. Whereas some studies report that individuals with AD can benefit from emotional content or context during their encoding, recall, or recognition, others presented contradictory findings demonstrating no such effects (Hamann et al., 2000; Kazui et al., 2000, 2003; Abrisqueta-Gomez et al., 2002; Boller et al., 2002; Kensinger et al., 2002; LaBar et al., 2005; Satler et al., 2007; Brueckner and Moritz, 2009; Perrin et al., 2012; Baran et al., 2014; Chainay et al., 2014; Kalenzaga et al., 2015; Gomez-Gallego and Gomez-Garcia, 2017). This inconsistency may stem from heterogeneity in participant characteristics (e.g., severity of dementia, cognitive profiles such as degree of attention, and deficits in executive functioning) in the current literature. However, more crucial differences between published reports include the nature of experimental conditions and tasks that comprise the study design, as previous studies relied heavily on non-standardized experimental procedures. For example, different types of stimuli were used including written words, recorded dialogues, images of natural or manufactured objects, and photos of living and non-living objects. In addition, a variety of experimental paradigms have been tested, including perceptual priming, immediate or delayed recall, and explicit memory paradigms like free or cued recall.

Demonstrating whether and how experimental paradigms impact emotional valence findings in AD, Sava et al. (2015) reported that individuals with AD may not show enhanced memory effects during a recognition task without deeper and richer encoding, but they show a positivity bias (better recognition of positive memory) when the stimuli were repeated and named before the recognition task for richer and deeper encoding experience. In addition, they found that retrieval cues and supports improved AD patients’ recall of positive images. Similarly, Kalenzaga et al. (2015) found that individuals with AD show no enhancement in recognition of emotional words compared to neutral words. However, their recollection of information, which requires more engagement of episodic memory, was better with emotional words. In this sense, experimental paradigms and conditions such as the encoding procedures, test forms of retrieval, and relevant strategies play a critical role in understanding the effects of emotional valence in language and cognitive processing in AD.

Further investigation is needed to tease apart the effects of experimental paradigms, especially during naming tasks, and to better understand the role that emotional valence can play during word retrieval, an ability that is substantially impaired from early on in AD (Garrard et al., 2005; Balthazar et al., 2008 Kavé and Goral, 2016, 2018). Using an experimental paradigm that does not place a heavy demand on memory encoding process, such as the use of verb fluency tasks, would provide a novel insight into the effects of emotional valence on retrieval of words by people experiencing AD. Verb fluency tasks require participants to name as many words as possible for things that people do in a certain time period, which does not require encoding during the experiment but place heavy emphasis on recall and retrieval of relevant lexical items (Woods et al., 2005; Alegret et al., 2018; Paek and Murray, 2021). Verbal fluency tasks are commonly utilized in both clinical and research settings for populations with AD, given their superior sensitivity in discovering qualitative differences in semantic, lexical, and phonological skills exhibited by an individual. However, it remains unclear whether the lexical items generated during verb fluency tasks would exhibit different levels of emotional valence in individuals with AD compared to cognitively healthy older adults (CHOA), due to the effect of emotional enhancement.

Therefore, the current study aimed to examine whether individuals with amnestic AD show increased emotional valence in their verb fluency responses compared to CHOA, since words with increased emotional valence may be easier to retrieve (Brueckner and Moritz, 2009 Perrin et al., 2012; Sava et al., 2015; Bohn et al., 2016). We predicted that emotional valence analysis would demonstrate a positivity bias given the findings of better recall or recognition of positive stimuli from some previous AD studies. Additionally, we hypothesized that the degree of emotional valence in words would correlate with the severity of verbal memory symptoms caused by dementia. Words with higher emotional valence may be more resilient to progressive cognitive declines, more resistant to neural deterioration, and therefore more easily accessed and retrieved in people with AD. This would be similar to the phenomenon that words with certain psycholinguistic properties (e.g., higher word frequency and lower age of acquisition) are more easily retrieved than others (Forbes-McKay et al., 2005; Vita et al., 2014; Rofes et al., 2019; Paek et al., 2020).

## Materials and Methods

### Participants

#### Inclusion/Exclusion Criteria

A total of 32 participants were enrolled in the current study, with 15 individuals who were diagnosed with mild to moderate amnestic AD and 17 individuals who were CHOA. All participants in this study were native speakers of English who were right-handed and who passed hearing and vision screening (either corrected or uncorrected). Hearing screening was administered using the speech discrimination subtest of the *Arizona Battery for Communication Disorders of Dementia* (ABCD; Bayles and Tomoeda, 1993), and vision was screened using a picture matching task. To be included in the study, all participants were required to score over 70% accuracy on the hearing screening test and 75% accuracy on the vision screening test. To meet the inclusionary criteria of the AD group, participants were required to: (a) be diagnosed by their neurologists with ADRDs and (b) demonstrate amnestic profiles according to the consensus criteria described by McKhann et al. (2011). Thus, the participants with AD exhibited the following clinical symptoms: (a) insidious onset of symptoms, (b) progressive deficits or worsening reported by caregivers or observation, (c) prominent memory deficits with at least one other domain of cognition being impaired, including attention or executive function, and (d) no other diagnosed neurological or neurodegenerative disorder that could explain these deficits, such as Lewy body dementia or strokes. Participants in the control group were required to demonstrate: (a) no history of or existing neurological or neurodegenerative diseases such as traumatic brain injury, strokes, or dementia, (b) no developmental language disorders such as dyslexia, Asperger’s syndrome, or specific language impairments, (c) no psychiatric disorders such as schizophrenia or clinical depression, and (d) no history of substance or alcohol abuse. Participants in the control group were also required to perform above the age- and education-matched cutoff scores on the *Mini-Mental State Exam*-2 (MMSE-2; Folstein et al., 2010) as suggested by Crum et al. (1993).

The two participant groups did not show any statistically significant differences in age (*t*=−1.99, *df*=30, *p*=0.055) or level of education (*t*=1.41, *df*=30, *p*=0.169). However, their MMSE-2 scores differed significantly (*t*=3.75, *df*=15.56, *p*=0.002; unequal variance assumed), with lower scores for individuals with AD than for the control participants (see Table 1). The AD group was comprised of 5 males and 10 females, while there were seven males and 10 females in the control group. The chi-square test indicated that there is no significant difference between groups in gender distribution, *X*^2^ (1, *N*=32)=0.209, *p*=0.647. The present study was reviewed and approved by The University of Tennessee Institutional Review Board. All participants were provided verbal and written information about the study and compensated monetarily for their participation. Either the participants or their legal representatives provided informed consent after reviewing the study procedures, potential risks, and possible benefits.

**Table 1:** Demographic information and verb fluency results.

Variable	CHOA group (*n*=17)	AD group (*n*=15)	*t* (*df*=30)
Age	73 (7.08)	78.6 (8.79)	−1.99
Gender	7 males, 10 females	5 males, 10 females	
Years in education	15.88 (2.55)	14.53 (2.88)	1.41
MMSE-2	27.82 (1.24)	22.93 (4.92)	3.75^*^
Verb fluency performance			
Number of correct responses	11.65 (3.16)	8.27 (3.63)	2.82^*^
Emotional valence	6.01 (0.42)	6.27 (0.23)	−2.18^*^

*Mean and SD in parentheses. CHOA, cognitively healthy older adults; AD, Alzheimer’s dementia; and MMSE, Mini-Mental State Exam-2*.

* *p<0.05*.

### Procedures

#### Verb Fluency Task

All participants performed a verb fluency task for a 30s period (Woods et al., 2005 Fernaeus et al., 2008; Alegret et al., 2018; Paek and Murray, 2021). The participants were instructed to name as many things as possible that people do, such as eat and smell. The verbal responses were audio-recorded and transcribed after the experiment, then scored as correct or incorrect. Correct responses included verbs that are relevant to what people do, while incorrect responses included repetition of previously spoken verbs or irrelevant words or utterances (e.g., uh, I do not know, hmm). These incorrect responses were excluded from the analysis of emotional valence of words.

#### Neuropsychological Test Battery

A battery of neuropsychological tests was administered to all participants to delineate their cognitive-linguistic profiles. The battery included: (a) *Scales of Cognitive and Communicative Ability for Neurorehabilitation* (SCCAN; Milman and Holland, 2003) to test orientation, attention, problem solving, reading, writing, and speech and reading comprehension, (b) *Test of Memory and Learning – Senior Edition* (TOMAL-SE; Reynolds and Voress, 2012) to measure immediate and delayed verbal and nonverbal memory and learning, (c) *Calibrated Ideational Fluency Assessment* (CIFA; Schretlen and Vannorsdall, 2010) to delineate verbal and nonverbal fluency and executive function skills, (d) Digit and Visual Memory Span subtests of the *Wechsler Memory Scale – Revised* (WMS-R; Wechsler, 1987) to examine visual and verbal short-term and working memory, (e) Pictorial Analogies and Geometric Categories subtests of the *Comprehensive Test of Nonverbal Intelligence* (CTONI-2; Hammill et al., 2009) to quantify nonverbal reasoning skills, and (f) the Expressive and Receptive versions of the *Vocabulary Assessment Scales* (Gerhardstein-Nader, 2013) to determine vocabulary size and naming skills. See Table 2 for the results. Among the 32 participants, two participants with dementia were unable to complete this battery due to scheduling conflicts.

**Table 2:** Results of neuropsychological test battery.

Neuropsychological test	CHOA group (*n*=17)	AD group (*n*=13)	t (*df*=28)
*Wechsler Memory Scale-Revised*			
Digit span forward raw score	9.29 (1.86)	7.92 (2.4)	1.77
Digit span backward raw score	7.35 (2.74)	4.85 (2.48)	2.59^*^
Digit span total raw score	16.65 (4.08)	12.77 (4.02)	2.60^*^
Visual memory span forward raw score	7.41 (1.5)	6.08 (1.5)	2.41^*^
Visual memory span backward raw score	6.82 (1.55)	5.31 (1.32)	2.83^*^
Visual memory span total raw score	14.24 (2.39)	11.38 (2.29)	3.30^*^
*Comprehensive Test of Nonverbal Intelligence-2*			
Pictorial analogies raw score	15.47 (7.46)	12.15 (5.94)	1.31
Geometric categories raw score	16.53 (4.58)	14.15 (3.46)	1.56
*Vocabulary Assessment Scales*			
Expressive raw score	170.76 (29.23)	146.92 (35.32)	2.02
Receptive raw score	178.88 (25.31)	158 (27.29)	2.17^*^
*Scales of Cognitive and Communicative Ability for Neurorehabilitation*			
Total raw score (max 121)	86.59 (8.46)	70.15 (15.45)	3.46^*^
Oral expression raw score	17.94 (1.78)	14.46 (4.58)	2.60^*^
Orientation raw score	11.82 (2.48)	10.31 (2.43)	1.67
Memory raw score	14.41 (4.06)	9.15 (4.85)	3.23^*^
Speech comprehension raw score	12.47 (1.23)	10.38 (2.29)	2.97^*^
Reading comprehension raw score	11.12 (1.45)	9.69 (2.46)	1.98
Writing raw score	6.59 (0.87)	5.38 (1.85)	2.17^*^
Attention raw score	14.82 (1.98)	11.08 (2.4)	4.69^*^
Problem solving raw score	21.24 (2.05)	16.69 (3.82)	3.89^*^
*Test of Memory and Learning – Senior Edition*			
Facial memory raw score	25.88 (4.96)	20.38 (6.17)	2.71^*^
Memory for stories raw score	20.24 (9.05)	10.31 (7.13)	3.25^*^
Word list learning raw score	32.12 (8.05)	23.85 (10.11)	2.50^*^
Visual sequential memory raw score	12.94 (7.29)	6.85 (4.91)	2.59^*^
Object recall raw score	44 (11.45)	28.15 (11.68)	3.73^*^
Memory for location raw score	67.88 (6.52)	35.23 (22.08)	5.16^*^
Facial memory delayed raw score	30.71 (3.55)	23.31 (4.35)	5.13^*^
Memory for stories del raw score	15.12 (9.31)	6.38 (6.35)	2.90^*^
Object recall delayed raw score	37.29 (4.18)	30.62 (8.81)	2.53^*^
CIFA total	20.94 (9.04)	15.54 (7.34)	1.76
Line violations	2 (2.48)	3.23 (3.22)	−1.19
Scribbles	0.18 (0.73)	0.46 (0.78)	−1.03
Nameable	0.88 (1.58)	1.31 (1.11)	−0.83
Total rule breaks	3 (3.28)	5 (3.96)	−1.51
Exact copies	0.24 (0.56)	0.92 (1.71)	−1.40
Rotations and mirror images	0.29 (0.69)	0.31 (0.63)	−0.06
Minimal variations	1.71 (2.02)	1.62 (2.43)	0.11
Total perseverative	1.82 (2.3)	2.85 (3.72)	−0.93
Unacceptable design	5.47 (3.54)	7.85 (4.53)	−1.62
Acceptable design	15.47 (9.03)	7.69 (6.8)	2.59^*^
Percent unacceptable	17.73 (17.18)	52.02 (28.29)	−4.12^*^

*CHOA, cognitively healthy older adults; AD, Alzheimer’s dementia; and CIFA, Calibrated Ideational Fluency Assessment*.

* *p<0.05*.

### Data Analysis

The correct responses during verb fluency tasks were analyzed for each participant with respect to emotional valence. The value of emotional valence was determined using norms provided by Warriner et al. (2013), where the emotional valence of 13,915 words were rated by 1,827 adult participants residing in the US. As these participants indicated how they felt after reading each stimulus word on a scale from 1 to 9, the values of emotional valence used in our analysis indicate affective valence (emotional response) than semantic valence (factual knowledge; see Itkes and Kron, 2019). The emotional valence values of each response were averaged for each individual; then, SPSS 25 statistical analysis software was used to conduct independent sample *t* tests to investigate whether the two groups differed in their verbal fluency responses in terms of emotional valence.

The two groups were also compared in terms of their cognitive and linguistic skills by comparing the raw scores of each test from the neuropsychological test battery. Pearson correlation coefficients were calculated to measure relationships between the emotional valence in their verb fluency responses and neuropsychological test scores related to verbal memory and learning. This was performed to determine whether the impairment level of these cognitive domains correlated with the degree of emotion observed in their word production. The test scores used for these correlations were Memory for Stories and Word List Learning of TOMAL-SE, as verbal episodic memory and learning are one of the cognitive domains that are most impaired and that demonstrate the earliest declines in the amnestic phenotype of AD (McKhann et al., 2011).

## Results

### Verb Fluency Results

The participants with AD produced a lower number of correct responses during the 30s verb fluency task compared to CHOA (see Table 1). The analysis of emotional valence revealed that individuals with AD produced words with higher emotional valence than CHOA. CHOA produced 11.65 words with the average emotional valence of 6.01, while individuals with AD produced 8.27 words with the average emotional valence 6.27. Statistical significance between the two groups (*p*<0.05) was confirmed.

### Cognitive-Linguistic Profiles

The participants with AD showed poorer performances compared to CHOA on a number of cognitive and linguistic subtests, as shown in Table 2. The participants with dementia performed worse than CHOA during short term and working memory tests (Digit Span Backward and Digit Span Total of WMS-R; Visual Memory Span Forward and Backward, and Visual Memory Span Total) and a receptive vocabulary test (VAS-R; total scores). The participants with AD demonstrated lower scores in Oral Expression, Memory, Speech Comprehension, Writing, Attention, Problem Solving of SCCAN compared to CHOA. They also exhibited poorer memory skills compared to CHOA during TOMAL-SE testing, including Facial Memory, Memory for Stories, Word List Learning, Visual Sequential Memory, Object Recall, Memory for Location, Facial Memory Delayed, Memory for Stories Delayed, and Object Recall Delayed. In the CIFA test, the total number of acceptable designs and percent unacceptable designs were smaller in the AD group than observed in the CHOA group.

### Correlation Results

The relationships among verbal learning and memory and the degree of emotional valence in verb fluency responses were determined using correlation analyses. The Pearson correlation coefficients indicated that there existed a significant negative relationship between verbal episodic memory (TOMAL-SE Memory for Stories) and emotional valence (*r*=−0.403, *p*=0.027). Higher emotional valence found in the verb fluency responses of the study participants were associated with lower verbal episodic memory skills in all participants. That is, the fewer components of a story that a participant could recall and reproduce, the higher emotional valence that was found in their verb fluency responses. Similarly, there was a negative correlation between the TOMAL-SE Word List Learning and emotional valence (*r*=−0.378, *p*=0.039), indicating that the fewer words a participant could learn from a list and recall, the higher emotional valence that was observed in their verb fluency responses.

## Discussion

The current study examined the degree of emotional valence in verb fluency responses in individuals with AD and CHOA to delineate the effect of emotional enhancement of memory in AD, independent of the experimental encoding procedures (Werheid et al., 2011; Klein-Koerkamp et al., 2012; Kalenzaga et al., 2015; Sava et al., 2015). The participants with AD produced a smaller number of verbs during verb fluency tasks and their verbs exhibited higher emotional valence than those produced by CHOA. In addition, the degree of emotional valence negatively correlated with verbal episodic memory skills in the participants, suggesting that the more severe one’s memory symptoms, the higher the emotional valence in their verb fluency responses.

It has been debated whether the emotional enhancement of memory effects are observed in individuals with AD or whether they can benefit from such effects for retrieval of information. Although AD results in limbic damages from the earlier stages of the disease and the amygdala plays a very important role in emotional processing, the results of many previous research studies suggested that emotional enhancement of memory effects is relatively well preserved in individuals with AD when using intentional encoding tasks (Hamann et al., 2000; Kazui et al., 2000, 2003; Mizuno et al., 2000; Boller et al., 2002; LaBar et al., 2005; Horínek et al., 2007; Satler et al., 2007; Brueckner and Moritz, 2009; Schultz et al., 2009; Sundstrøm, 2011; Werheid et al., 2011; Perrin et al., 2012; Guzmán-Vélez et al., 2014; Kalenzaga et al., 2015; Bohn et al., 2016; Gomez-Gallego and Gomez-Garcia, 2017). The current study strengthens the evidence that emotional enhancement of memory is preserved in individuals with AD, since our participants retrieved words during verb fluency tasks that exhibited higher emotional valence than did that of the CHOA. In contrast to previous studies, our experimental paradigm did not require intentional (or implicit) encoding of stimuli during the experiment since it employed a verb fluency task. As this task does not involve encoding or learning new words or stimuli, there was little to no experimental influence on testing the emotional enhancement of memory effects. Instead, the participants were tested solely on retrieval of lexical items, i.e., searching through their mental lexicon to select and retrieve verbs that are relevant to the task instructions (i.e., name as many words as possible for things that people do). Accordingly, our findings indicated that the processes of search and retrieval for words as well as that of encoding in the memory system are affected by emotional factors in AD.

As emotion heavily influences word processing in healthy populations, for example, resulting in faster or more accurate recognition or retrieval of words with more emotional stimuli or contexts, our investigation of emotional valence of words produced by individuals with AD may provide insights not only into the cognitive changes due to disease progression, but also whether this factor can be leveraged for rehabilitation of word retrieval deficits in AD (Kuperman et al., 2014; Kauschke et al., 2019). Supporting our first hypothesis and consistent with the previous studies that showed positivity biases in memory recall in AD (e.g., Hamann et al., 2000; Brueckner and Moritz, 2009 Gallo et al., 2010; Perrin et al., 2012; Kalenzaga et al., 2015; Sava et al., 2015; Bohn et al., 2016), our participants with AD produced verbs with more pleasantness (i.e., higher emotional valence) during verb fluency tasks than did the CHOA. Our second hypothesis was also supported, with the data suggesting that higher emotional valence in verb fluency responses is associated with poorer immediate verbal memory skills. These findings may indicate that those words with higher emotional valence are easier to retrieve among the competing lexical items. Alternatively, it is also plausible that those words are more resilient to the effects of the cognitive and linguistic declines that are associated with AD and thus are preserved in the mental lexicon better or longer. Indeed, previous studies suggest that individuals with dementia can maintain or retrieve certain words for a longer period of time over the course of their disease progression, including those words with stronger or more salient psycholinguistic properties (e.g., word frequency, concreteness, imageability, and neighborhood density; Forbes-McKay et al., 2005; Vita et al., 2014; Rofes et al., 2019; Paek et al., 2020). However, further research is warranted to delineate whether this effect is attributable to stronger representations or easier access. That is, are words with higher emotional valence better preserved as they have stronger representations and connections in the mental lexicon, or among competing lexical items, are they more easily accessible during search and retrieval because of their psycholinguistic salience?

Most of the previous studies investigated the effects of emotion on retrieval using the category of objects or nouns (e.g., Brueckner and Moritz, 2009 Giffard et al., 2015; Rodríguez-Ferreiro et al., 2019). However, the current study suggests that these effects may be observed across different parts of speech, including verbs. As individuals with AD exhibits difficulties with production and comprehension of both nouns and verbs (Kim and Thompson, 2004; De Almeida et al., 2021), the findings in the current study might provide insights into development of treatment methods or to determine which words should be prioritized for treatment and maintenance. Future research should also examine whether this finding can be replicated across different types of verbal fluency tasks such as category fluency (e.g., supermarket items, animals) and letter fluency (e.g., words that start with the letter H). The verb fluency task requires retrieval of human actions only, which may be more pertinent to memories about self (self-concept and self-continuity; Rasmussen and Berntsen, 2009) or autobiographical memory. Comparison among traditional category and letter fluency tasks and verb fluency tasks may provide additional insights into the cognitive changes in AD.

The limitations and future directions of the current study include the following. Since only those individuals with mild to moderate dementia were tested in the study, testing the hypotheses with individuals demonstrating different severity levels would offer a more comprehensive examination of the effects. It has been suggested that retrieval of emotional information in AD is enhanced by amygdala and medial temporal lobe structures engaging in the process, even when these regions are impaired (Klein-Koerkamp et al., 2012). However, as the functional impairments caused by neuropathological changes in limbic regions will only be worsened in those experiencing more severe AD, it is plausible that emotional enhancement of memory effects may not be observed or may be attenuated in patients exhibiting symptoms of more severe AD. Thus, future studies will be needed to examine whether the effects of emotional valence differ across the continuum of disease, from individuals with mild cognitive impairments to those experiencing more severe dementia.

Since we used a 30-s verb fluency task to probe emotional valence in words produced by our participants (Fernaeus et al., 2008), it is possible that the psycholinguistic properties of words might differ if the participants were required to perform the task for a 1-min period, because the psycholinguistic quality of words in verbal fluency performance changes over time (Vonk et al., 2019). That is, different levels of emotional valence might have been observed if the participants were to have put forth effort beyond the 30-s period we used. Future studies will need to examine whether there exist differences in the emotional valence during the initial time bins (e.g., 0–30s) versus later ones (e.g., 30–60s) and to investigate what might provide more sensitive information about the cognitive status of individuals with AD.

In addition, the sample size of the current study was relatively small, which might be associated with reduced statistical power and difficulties with generalization of findings to the population of interest. It is also possible that there are disparities between different race and ethnicity groups in terms of what is considered positive or negative emotional valence (i.e., the ratings of the emotional valence) stemming from the cultural differences. The distribution of race and ethnicity in our sample was mostly homogeneous, including only one black participant with AD and all the others being white. However, the normative study by Warriner et al. (2013) that we used to measure emotional valence of generated words did not present the race and ethnicity data of their sample. Moreover, race and ethnicity as well as socioeconomic status may be a factor that influences linguistic performance during word retrieval and verbal fluency (Johnson-Selfridge et al., 1998; Gladsjo et al., 1999). Thus, future studies will need to carefully consider these demographic characteristics of the participant sample as well as the normative sample.

In summary, the current study provides insights into the effects of emotion on word retrieval processes in AD, as we found that word retrieval may be easier or more challenging depending on the emotional aspect of the lexical item. It also supports the hypothesis that lexical items with higher emotional valence may be more resilient to the cognitive declines and may be a good candidate for treatment or maintenance. That is, in selecting words for rehabilitation or in choosing which words should be practiced for maintenance in AD, the emotional valence of words may play an important role in leveraging the outcomes.

## Data Availability Statement

The raw data supporting the conclusions of this article will be made available by the authors, without undue reservation.

## Ethics Statement

The studies involving human participants were reviewed and approved by University of Tennessee Institutional Review Board. The patients/participants provided their written informed consent to participate in this study.

## Author Contributions

EP designed and performed the experiments, analyzed the data, and wrote the manuscript.

## Funding

This work was funded in part by Alzheimer’s Association Greater Indiana Chapter.

## Conflict of Interest

The author declares that the research was conducted in the absence of any commercial or financial relationships that could be construed as a potential conflict of interest.

## Publisher’s Note

All claims expressed in this article are solely those of the authors and do not necessarily represent those of their affiliated organizations, or those of the publisher, the editors and the reviewers. Any product that may be evaluated in this article, or claim that may be made by its manufacturer, is not guaranteed or endorsed by the publisher.

## References

[ref1] Abrisqueta-Gomez J. Bueno O. Oliveira M. Bertolucci P. (2002). Recognition memory for emotional pictures in Alzheimer’s patients. Acta Neurol. Scand. 105, 51–54. doi: 10.1034/j.1600-0404.2002.00035.x 11903109

[ref2] Alegret M. Peretó M. Pérez A. Valero S. Espinosa A. Ortega G.. (2018). The role of verb fluency in the detection of early cognitive impairment in Alzheimer’s disease. J. Alzheimers Dis. 62, 611–619. doi: 10.3233/JAD-170826, PMID: 29480180

[ref3] Alzheimer’s Association (2021). Alzheimer's disease facts and figures. Alzheimers Dement. 17, 1–104. 10.1002/alz.12328 33756057

[ref001] Balthazar M. L. F. Cendes F. G. Damasceno B. P. (2008). Semantic error patterns on the Boston Naming Test in normal aging, amnestic mild cognitive impairment, and mild Alzheimer’s disease: Is there semantic disruption? Neuropsychology 22, 703–709. 18999343 10.1037/a0012919

[ref4] Baran Z. Cangöz B. Ozel-Kizil E. T. (2014). The impact of aging and Alzheimer’s disease on emotional enhancement of memory. Eur. Neurol. 72, 30–37. doi: 10.1159/000359924, PMID: 24853408

[ref5] Bayles K. Tomoeda C. (1993). Arizona Battery for Communication Disorders of Dementia. Tucson, AZ: Canyonlands. 10.3109/13682829609042219 8776438

[ref6] Blessing A. Keil A. Linden D. E. J. Heim S. Ray W. J. (2006). Acquisition of affective dispositions in dementia patients. Neuropsychologia 44, 2366–2373. doi: 10.1016/j.neuropsychologia.2006.05.004 16777148

[ref7] Bohn L. Kwong See S. T. Fung H. H. (2016). Time perspective and positivity effects in Alzheimer’s disease. Psychol. Aging 31, 574–582. doi: 10.1037/pag0000084, PMID: 26974590

[ref8] Boller F. El Massioui F. Devouche E. Traykov L. Pomati S. Starkstein S. E. (2002). Processing emotional information in Alzheimer’s disease: effects on memory performance and neurophysiological correlates. Dement. Geriatr. Cogn. Disord. 14, 104–112. doi: 10.1159/000064932, PMID: 12145458

[ref9] Broster L. S. Blonder L. X. Jiang Y. (2012). Does emotional memory enhancement assist the memory-impaired? Front. Aging Neurosci. 4:2. doi: 10.3389/fnagi.2012.00002 22479245 PMC3315887

[ref10] Brueckner K. Moritz S. (2009). Emotional valence and semantic relatedness differentially influence false recognition in mild cognitive impairment, Alzheimer’s disease, and healthy elderly. J. Int. Neuropsychol. Soc. 15, 268–276. doi: 10.1017/S135561770909047X, PMID: 19203441

[ref11] Chainay H. Sava A. Michael G. A. Landre L. Versace R. Krolak-Salmon P. (2014). Impaired emotional memory enhancement on recognition of pictorial stimuli in Alzheimer’s disease: no influence of the nature of encoding. Cortex 50, 32–44. doi: 10.1016/j.cortex.2013.10.001, PMID: 24268828

[ref12] Crum R. M. Anthony J. C. Bassett S. S. Folstein M. F. (1993). Population-based norms for the mini-mental state examination by age and educational level. JAMA 269, 2386–2391. doi: 10.1001/jama.1993.03500180078038, PMID: 8479064

[ref13] De Almeida R. G. Mobayyen F. Antal C. Kehayia E. Nair V. P. Schwartz G. (2021). Category-specific verb-semantic deficits in Alzheimer’s disease: evidence from static and dynamic action naming. Cogn. Neuropsychol. 38, 1–26. doi: 10.1080/02643294.2020.1858772, PMID: 33455543

[ref14] De Wit L. Marsiske M. O’Shea D. Kessels R. P. C. Kurasz A. M. DeFeis B.. (2021). Procedural learning in individuals with amnestic mild cognitive impairment and Alzheimer’s dementia: a systematic review and meta-analysis. Neuropsychol. Rev. 31, 103–114. doi: 10.1007/s11065-020-09449-1, PMID: 32897482 PMC7889687

[ref15] Di Marco L. Y. Marzo A. Muñoz-Ruiz M. Ikram M. A. Kivipelto M. Ruefenacht D.. (2014). Modifiable lifestyle factors in dementia: a systematic review of longitudinal observational cohort studies. J. Alzheimers Dis. 42, 119–135. doi: 10.3233/JAD-132225, PMID: 24799342

[ref16] Fernaeus S. E. Östberg P. Hellström Å. Wahlund L. O. (2008). Cut the coda: early fluency intervals predict diagnoses. Cortex 44, 161–169. doi: 10.1016/j.cortex.2006.04.002, PMID: 18387545

[ref17] Folstein M. F. Folstein S. E. McHugh P. R. Fanjiang G. (2010). Mini-Mental State Examination: MMSE-2. Lutz, FL: Psychological Assessment Resources.

[ref18] Forbes-McKay K. E. Ellis A. W. Shanks M. F. Venneri A. (2005). The age of acquisition of words produced in a semantic fluency task can reliably differentiate normal from pathological age related cognitive decline. Neuropsychologia 43, 1625–1632. doi: 10.1016/j.neuropsychologia.2005.01.008, PMID: 16009244

[ref19] Gallo D. A. Foster K. T. Wong J. T. Bennett D. A. (2010). False recollection of emotional pictures in Alzheimer’s disease. Neuropsychologia 48, 3614–3618. doi: 10.1016/j.neuropsychologia.2010.08.011, PMID: 20727904 PMC2949550

[ref20] Garrard P. Maloney L. M. Hodges J. R. Patterson K. (2005). The effects of very early Alzheimer’s disease on the characteristics of writing by a renowned author. Brain 128, 250–260. doi: 10.1093/brain/awh341, PMID: 15574466

[ref21] Gerhardstein-Nader R. (2013). Vocabulary Assessment Scales. Psychological Assessment Resources. Lutz, FL: PAR.

[ref22] Giffard B. Laisney M. Desgranges B. Eustache F. (2015). An exploration of the semantic network in Alzheimer’s disease: influence of emotion and concreteness of concepts. Cortex 69, 201–211. doi: 10.1016/j.cortex.2015.05.020, PMID: 26094148

[ref23] Gladsjo J. A. Schuman C. C. Evans J. D. Peavy G. M. Miller S. W. Heaton R. K. (1999). Norms for letter and category fluency: demographic corrections for age, education, and ethnicity. Assessment 6, 147–178. doi: 10.1177/107319119900600204, PMID: 10335019

[ref24] Gomez-Gallego M. Gomez-Garcia J. (2017). Negative bias in the perception and memory of emotional information in Alzheimer disease. J. Geriatr. Psychiatry Neurol. 30, 131–139. doi: 10.1177/0891988716686833, PMID: 28421897

[ref25] Guzmán-Vélez E. Feinstein J. S. Tranel D. (2014). Feelings without memory in Alzheimer disease. Cogn. Behav. Neurol. 27, 117–129. doi: 10.1097/WNN.0000000000000020, PMID: 25237742 PMC4175156

[ref26] Hamann S. B. Monarch E. S. Goldstein F. C. (2000). Memory enhancement for emotional stimuli is impaired in early Alzheimer’s disease. Neuropsychology 14, 82–92. doi: 10.1037/0894-4105.14.1.82, PMID: 10674800

[ref27] Hammill D. D. Pearson N. A. Weiderholt J. L. (2009). Comprehensive Test of Nonverbal Intelligence. 2nd *Edn.* Austin, TX: Pro-Ed.

[ref28] Harrison B. E. Son G. R. Kim J. Whall A. L. (2007). Preserved implicit memory in dementia: a potential model for care. Am. J. Alzheimers Dis. Other Dement. 22, 286–293. doi: 10.1177/1533317507303761, PMID: 17712159 PMC10846121

[ref29] Horínek D. Varjassyová A. Hort J. (2007). Magnetic resonance analysis of amygdalar volume in Alzheimer’s disease. Curr. Opin. Psychiatry 20, 273–277. doi: 10.1097/YCO.0b013e3280ebb613, PMID: 17415082

[ref30] Itkes O. Kron A. (2019). Affective and semantic representations of valence: a conceptual framework. Emot. Rev. 11, 283–293. doi: 10.1177/1754073919868759

[ref31] Johnson-Selfridge M. T. Zalewski C. Aboudarham J. F. (1998). The relationship between ethnicity and word fluency. Arch. Clin. Neuropsychol. 13, 319–325. doi: 10.1093/arclin/13.3.319, PMID: 14590645

[ref32] Kalenzaga S. Piolino P. Clarys D. (2015). The emotional memory effect in Alzheimer’s disease: Emotional words enhance recollective experience similarly in patients and control participants. Cogn. Emot. 29, 342–350. 24734952 10.1080/02699931.2014.907127

[ref33] Kauschke C. Bahn D. Vesker M. Schwarzer G. (2019). The role of emotional valence for the processing of facial and verbal stimuli positivity or negativity bias? Front. Psychol. 10:1654. doi: 10.3389/fpsyg.2019.01654, PMID: 31402884 PMC6676801

[ref34] Kavé G. Goral M. (2016). Word retrieval in picture descriptions produced by individuals with Alzheimer’s disease. J. Clin. Exp. Neuropsychol. 38, 958–966. doi: 10.1080/13803395.2016.1179266 27171756 PMC4983450

[ref35] Kavé G. Goral M. (2018). Word retrieval in connected speech in Alzheimer’s disease: a review with meta-analyses. Aphasiology 32, 4–26. doi: 10.1080/02687038.2017.1338663

[ref36] Kazui H. Mori E. Hashimoto M. Hirono N. (2003). Enhancement of declarative memory by emotional arousal and visual memory function in Alzheimer’s disease. J. Neuropsychiatr. Clin. Neurosci. 15, 221–226. doi: 10.1176/jnp.15.2.221, PMID: 12724465

[ref37] Kazui H. Mori E. Hashimoto M. Hirono N. Imamura T. Tanimukai S.. (2000). Impact of emotion on memory: controlled study of the influence of emotionally charged material on declarative memory in Alzheimer’s disease. Br. J. Psychiatry 177, 343–347. doi: 10.1192/bjp.177.4.343 11116776

[ref38] Kensinger E. A. Brierley B. Medford N. Growdon J. H. Corkin S. (2002). Effects of normal aging and Alzheimer’s disease on emotional memory. Emotion 2, 118–134. doi: 10.1037/1528-3542.2.2.118 12899186

[ref39] Kim M. Thompson C. K. (2004). Verb deficits in Alzheimer’s disease and agrammatism: implications for lexical organization. Brain Lang. 88, 1–20. doi: 10.1016/S0093-934X(03)00147-0, PMID: 14698726 PMC3079403

[ref40] Klein-Koerkamp Y. Baciu M. Hot P. (2012). Preserved and impaired emotional memory in Alzheimer’s disease. Front. Psychol. 3:331. doi: 10.3389/fpsyg.2012.00331 23049516 PMC3442282

[ref41] Kuperman V. Estes Z. Brysbaert M. Warriner A. B. (2014). Emotion and language: valence and arousal affect word recognition. J. Exp. Psychol. Gen. 143, 1065–1081. doi: 10.1037/a0035669, PMID: 24490848 PMC4038659

[ref42] LaBar K. S. Torpey D. C. Cook C. A. Johnson S. R. Warren L. H. Burke J. R.. (2005). Emotional enhancement of perceptual priming is preserved in aging and early-stage Alzheimer’s disease. Neuropsychologia 43, 1824–1837. doi: 10.1016/j.neuropsychologia.2005.01.018, PMID: 16154458

[ref43] McKhann G. M. Knopman D. S. Chertkow H. Hyman B. T. Jack C. R. Jr. Kawas C. H.. (2011). The diagnosis of dementia due to Alzheimer’s disease: recommendations from the national institute on aging Alzheimer’s association workgroups on diagnostic guidelines for Alzheimer’s disease. Alzheimers Dement. 7, 263–269. doi: 10.1016/j.jalz.2011.03.005, PMID: 21514250 PMC3312024

[ref44] Milman L. Holland A. (2003). Scales of Cognitive and Communicative Ability for Neurorehabilitation. Pro-Ed. Tucson, USA: The University of Arizona.

[ref45] Mizuno K. Wakai M. Takeda A. Sobue G. (2000). Medial temporal atrophy and memory impairment in early stage of Alzheimer’s disease: an MRI volumetric and memory assessment study. J. Neurol. Sci. 173, 18–24. doi: 10.1016/S0022-510X(99)00289-0, PMID: 10675575

[ref46] Nashiro K. Mather M. (2011). The effect of emotional arousal on memory binding in normal aging and Alzheimer’s disease. Am. J. Psychol. 124, 301–312. doi: 10.5406/amerjpsyc.124.3.0301, PMID: 21977692 PMC3312600

[ref47] Oren S. Willerton C. Small J. (2014). Effects of spaced retrieval training on semantic memory in Alzheimer’s disease: a systematic review. J. Speech Lang. Hear. Res. 57, 247–270. doi: 10.1044/1092-4388(2013/12-0352), PMID: 24023380

[ref48] Paek E. J. Murray L. L. (2021). Quantitative and qualitative analysis of verb fluency performance in individuals with probable Alzheimer’s disease and healthy older adults. Am. J. Speech Lang. Pathol. 30, 481–490. doi: 10.1044/2019_AJSLP-19-00052, PMID: 32551834

[ref49] Paek E. J. Murray L. L. Newman S. D. (2020). Neural correlates of verb fluency performance in cognitively healthy older adults and individuals with dementia: a pilot fMRI study. Front. Aging Neurosci. 12:73. doi: 10.3389/fnagi.2020.00073, PMID: 32265685 PMC7100367

[ref50] Perrin M. Henaff M. A. Padovan C. Faillenot I. Merville A. Krolak-Salmon P. (2012). Influence of emotional content and context on memory in mild Alzheimer’s disease. J. Alzheimers Dis. 29, 817–826. doi: 10.3233/JAD-2012-111490, PMID: 22349683

[ref51] Rasmussen A. S. Berntsen D. (2009). Emotional valence and the functions. Mem. Cogn. 37, 477–492. doi: 10.3758/MC.37.4.477, PMID: 19460954

[ref52] Reynolds C. R. Voress J. K. (2012). Test of Memory and Learning–Senior Edition Examiner’s Manual. Austin, TX: PRO-ED Inc..

[ref53] Rodríguez-Ferreiro J. Martínez C. Cuetos F. (2019). Differential effects of negative and positive emotional content over veridical and false recognition in aging and Alzheimer’s disease. J. Neurolinguistics 49, 109–116. doi: 10.1016/j.jneuroling.2018.10.001

[ref54] Rofes A. de Aguiar V. Ficek B. Wendt H. Webster K. Tsapkini K. (2019). The role of word properties in performance on fluency tasks in people with primary progressive aphasia. J. Alzheimers Dis. 68, 1521–1534. doi: 10.3233/JAD-180990, PMID: 30909222 PMC6548439

[ref55] Satler C. Garrido L. M. Sarmiento E. P. Leme S. Conde C. Tomaz C. (2007). Emotional arousal enhances declarative memory in patients with Alzheimer’s disease. Acta Neurol. Scand. 116, 355–360. doi: 10.1111/j.1600-0404.2007.00897.x, PMID: 17986092

[ref56] Sava A. A. Paquet C. Krolak-Salmon P. Dumurgier J. Hugon J. Chainay H. (2015). Emotional memory enhancement in respect of positive visual stimuli in Alzheimer’s disease emerges after rich and deep encoding. Cortex 65, 89–101. doi: 10.1016/j.cortex.2015.01.002, PMID: 25681651

[ref57] Schretlen D. J. Vannorsdall T. D. (2010). Calibrated Ideational Fluency Assessment (CIFA) Professional Manual. Lutz, Florida: Psychological Assessment Resources, Inc.

[ref58] Schultz R. R. De Castro C. C. Bertolucci P. H. F. (2009). Memory with emotional content, brain amygdala and Alzheimer’s disease. Acta Neurol. Scand. 120, 101–110. doi: 10.1111/j.1600-0404.2008.01132.x, PMID: 19154540

[ref59] Sundstrøm M. (2011). Modeling recall memory for emotional objects in Alzheimer’s disease. Aging Neuropsychol. Cognit. 18, 396–413. doi: 10.1080/13825585.2011.567324, PMID: 21728888

[ref60] van Dulmen S. Smits L. Eide H. (2017). Filling in memory gaps through emotional communication: promising pathways in caring for persons with dementia. Patient Educ. Couns. 100, 2121–2124. doi: 10.1016/j.pec.2017.06.014, PMID: 28641992

[ref61] Vita M. G. Marra C. Spinelli P. Caprara A. Scaricamazza E. Castelli D.. (2014). Typicality of words produced on a semantic fluency task in amnesic mild cognitive impairment: linguistic analysis and risk of conversion to dementia. J. Alzheimers Dis. 42, 1171–1178. doi: 10.3233/JAD-140570, PMID: 25024315

[ref62] Vonk J. M. J. Flores R. J. Rosado D. Qian C. Cabo R. Habegger J.. (2019). Semantic network function captured by word frequency in nondemented APOE ε4 carriers. Neuropsychology 33, 256–262. doi: 10.1037/neu0000508, PMID: 30489116 PMC6466625

[ref63] Warriner A. B. Kuperman V. Brysbaert M. (2013). Norms of valence, arousal, and dominance for 13,915 English lemmas. Behav. Res. Methods 45, 1191–1207. doi: 10.3758/s13428-012-0314-x, PMID: 23404613

[ref64] Wechsler D. (1987). Wechsler Memory Scale–Revised. The Psychological Corporation. San Antonio, TX Psychological Corporation.

[ref65] Werheid K. McDonald R. S. Simmons-Stern N. Ally B. A. Budson A. E. (2011). Familiar smiling faces in Alzheimer’s disease: understanding the positivity-related recognition bias. Neuropsychologia 49, 2935–2940. doi: 10.1016/j.neuropsychologia.2011.06.022, PMID: 21736891 PMC3748937

[ref66] Woods S. P. Scott J. C. Sires D. A. Grant I. Heaton R. K. Tröster A. I.. (2005). Action (verb) fluency: test–retest reliability, normative standards, and construct validity. J. Int. Neuropsychol. Soc. 11, 408–415. doi: 10.1017/S1355617705050460, PMID: 16209421

[ref67] Wright C. I. Dickerson B. C. Feczko E. Negeira A. Williams D. (2007). A functional magnetic resonance imaging study of amygdala responses to human faces in aging and mild Alzheimer’s disease. Biol. Psychiatry 62, 1388–1395. doi: 10.1016/j.biopsych.2006.11.013, PMID: 17336945

[ref68] Yates L. A. Ziser S. Spector A. Orrell M. (2016). Cognitive leisure activities and future risk of cognitive impairment and dementia: systematic review and meta-analysis. Int. Psychogeriatr. 28, 1791–1806. doi: 10.1017/S1041610216001137, PMID: 27502691

